# Serologic Survey of the Pandemic H1N1 2009 Virus in Guangdong Province, China: A Cross Sectional Study

**DOI:** 10.1371/journal.pone.0023034

**Published:** 2011-08-10

**Authors:** Xin Zhang, Jianfeng He, Linghui Li, Xiaolan Zhu, Changwen Ke, Hanzhong Ni, Nianmei Hou, Haojie Zhong, Jie Wu

**Affiliations:** Center for Disease Control and Prevention of Guangdong Province, Guangzhou, People's Republic of China; University of Georgia, United States of America

## Abstract

**Background:**

Relying on surveillance of clinical cases limits the ability to understand the full impact and severity of an epidemic, which urges a deep insight into the serological evidence of infection and transmission feature of pandemic H1N1 2009 (pH1N1) virus in Guangdong province.

**Methods:**

In this cross-sectional serological survey, serum samples were collected by multi-stage stratified random sampling in Jan 2010. Antibody titers were measured by hemagglutination inhibition (HI) assay. Age-specific and region-specific prevalence were calculated based on the results of HI assay (positive, HI titer≥1∶40).

**Results:**

A total of 4,319 serum samples had been collected from subjects without vaccination with pH1N1 vaccine. The seroprevalence was 22.82% (985/4,319). By contrast, there was a marked spatial heterogeneity in prevalence. The seroprevalence was 27.3% in large city, 21.4% in medium cities, higher than that of 20.2% in rural areas. The seroprevalence was highest in 11–20 age group (32.8%), however, in those above 60 years of age group, which was 12.6%, lower than other age groups. On the other hand, antibody titers to pH1N1 virus were highest in school children, which were followed by a gradual decrease in adult. However, in the elderly groups from cities, especially from large city, the antibody titer to pH1N1 increased significantly and reached a much higher level.

**Conclusion:**

Our results showed that the prevalence for pH1N1 was correlated with age and population density. Preexisting antibody may have protected the very old from pH1N1 infection, while original antigenic sin and immunosenescence may have contributed to greater severity once infected. These should be considered when studying the pathogenesis and transmission of influenza virus and formulating strategies on vaccination and treatment.

## Introduction

In March 2009, there was an unexpected increase in Influenza-Like Illness (ILI), with a total of 854 cases of pneumonia and 59 deaths in Mexico. A novel Influenza virus, now called pandemic H1N1 2009 (pH1N1), was subsequently found to be a reassortant derived from influenza viruses of four origins: classical swine, European swine, avian, and human influenza viruses [1]. The lack of neutralizing antibodies to the new virus allowed rapid spread around the world [2]–[4].

Guangdong, a province on the southern coast of China, has 21 cities with registering 95.44 million permanent residents. Guangdong was often considered to play a significant role in influenza H3N2 transmission [5]. On May 18, 2009 the first case of pH1N1 was detected by Guangdong CDC in Guangdong province. The epidemic in Guangdong reached its peak in November, and attenuated to baseline levels by late December, 2009. By the end of the year 2009, according to the provincial sentinel surveillance data, 9,784 cases of pH1N1, including 451 serious cases and 36 death cases, were confirmed by real-time PCR in Guangdong. The epidemiology of pH1N1 appeared to be a mild to moderate disease affecting school-age children preferentially over older adults. In10–20 years of age group, the morbidity rate was 53.20% (5205/9784), the serious cases morbidity rate was 1.4%(73/5205) and the case fatality rate was 0.12% (6/5205), however, in above 60 years of age group, which were 0.34%(33/9784), 30.3% (10/33) and 6.06% (2/33), respectively [6], [7]. The case fatality rate of pH1N1was the highest in elderly adults, unlike during the 1918–1919 pandemic which was greatest in the young [8].

In fact, relying on surveillance of clinical cases limits the ability to understand the full impact and severity of an epidemic, which urges a deep insight into the serological evidence of infection and transmission feature of pH1N1 in Guangdong. At the end of the winter epidemic wave, we conducted a seroepidemiologic cross sectional study among general population in Guangdong. This study aimed to describe the characterization of serologic differences among various age groups and to understand the epidemiological feature of pH1N1 which may provide valuable information for study on the pathogenesis and transmission of influenza virus and formulating strategies on vaccination and treatment.

## Results

### 1. Antibody responses by gender, geographical distribution, occupation and symptoms ever appeared in last 6 months

A total of 4,319 serum samples had been collected from subjects without vaccination with pH1N1 vaccine during 11–22 Jan 2010. Those serum samples, distributed in 21 cities, 25 counties, 85 streets or townships, 144 residential areas, were tested by HI, with an overall seroprevalence as 22.82%(985/4,319). Based on the population data of Guangdong province which was provided by National Bureau of Statistics of China, the adjusted seroprevalence was 18.0% (95%CI, 16.5–19.5%). The post population adjusted weight coefficient was calculated by the formula:
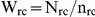

**Nrc: the number of persons in different regions, gender, age groups in the general population, Guangdong; nrc: the number of subjects in different regions, gender, age groups in the sampling. Where r represents region, r = 1 represents large cities, r = 2 represents medium cities, r = 3 represents rural areas; c represents gender, age groups, c = 1 represents male, 0–5, c = 2 represents male, 6–15, …, c = 10 represents female, ≥60.** There were registering 95.44 million permanent residents in Guangdong. Therefore, the estimate of natural infection population was 17.18 million.

The seroprevalences of pH1N1 in various groups of populations were shown in Table 1. The seroprevalences were22.2% in male and 23.5% in female. There were no statistically significant differences in gender. (p>0.05)

**Table 1:** Antibody response against pH1N1among different groups.

Variable	Groups	All, No.	No.positive (Seroprevalence %)	*p*
Gender	Male	2158	478(22.2)	0.304
	Female	2161	507(23.5)	
Age	0–5	752	212(28.2)	0.000
	6–15	992	284(28.6)	
	16–24	820	240(29.3)	
	25–60	830	132(15.9)	
	>60	925	117(12.6)	
Region	Large cities	1331	364(27.3)	0.000
	Medium cities	1543	330(21.4)	
	Rural areas	1445	291(20.1)	
Occupation	Children in household	245	59(24.1)	0.000
	Children in nursery	757	198(26.2)	
	Students in school	1169	386(33.0)	
	Teachers	139	31(22.3)	
	Health care staff	105	35(33.3)	
	Others	1902	276(14.5)	
Symptoms	Fever	1218	334(27.4)	0.000
	Cough	1912	462(24.2)	0.060
	Sore throat	1506	366(24.3)	0.090
	ILI	1068	297(27.8)	0.000
Seasonal influenza vaccination	Post- vaccination	461	119(25.8)	0.105
	Non- vaccination	3858	866(22.4)	

In total serum samples, there were 1,331 samples from large cities, 1,543 samples from medium cities and 1,445 samples from rural areas. By contrast, there was a marked spatial heterogeneity in seroprevalences, with 27.3% in large cities, 21.4% in medium cities, 20.1% in rural areas, respectively (p<0.001).

Among various groups of occupation, the seroprevalences were 33.3% in health care staff and 33.0% in students, much higher than that of the others jobs (p<0.001).

The seroprevalences were 27.4% (334/1218) in subjects with fever which appeared in last 6 months, 24.2%(462/1912) with cough, 24.3% (366/1506) with sore throat, and 27.8%(297/1068) with ILI (fever plus cough or sore throat). However, the seroprevalence was 20.96% (426/2032) in subjects without any symptoms mentioned above.

Our data showed that there was no significant difference in seroprevalence against pH1N1 with or without vaccination seasonal trivalent influenza vaccination (TIV) (p>0.05).

### 2. Antibody responses by age

The seroprevalences of pH1N1 in all five age groups of populations were shown in Table 1. To enable finer age assessment in subjects, especially in the elderly, original five age strata (0–5, 6–15, 16–24, 25–59, and 60∼) were subdivided into 0–5, 6–10, 11–20, 21–30, 31–40, 41–50, 51–60, 61–70, 71–80, >81.(Table 2.)

**Table 2:** Sample size, seroprevalences and GMTs in different age groups.

Age group	No. of subjects	Seroprevalence %	Geometric mean titer (95%CI)	*p*
0–5	752	28.2	11.77(10.77–12.86)	0.000
6–10	640	28.3	12.65(11.46–13.96)	
11–20	799	32.8	14.7(13.4–16.13)	
21–30	597	20.5	10.02(9.08–11.06)	
31–40	271	16.6	9.05(7.83–10.45)	
41–50	217	15.2	8.44(7.27–9.79)	
51–60	171	13.5	7.93(6.84–9.2)	
61–70	613	12.1	7.81(7.18–8.49)	
71–80	201	12.4	7.53(6.6–8.6)	
>80	58	13.8	7.32(5.85–9.17)	

Seroprevalence of pH1N1 was highest in school children, especially those of 11–20 years old, among whom 32.8% had HI titers≥40 to H1N1. There was a very noticeable trend to decrease in seroprevalence in adults. However, seroprevalence in those≥80 years of age increased slightly, arriving at 13.8%.

**Figure 1:**
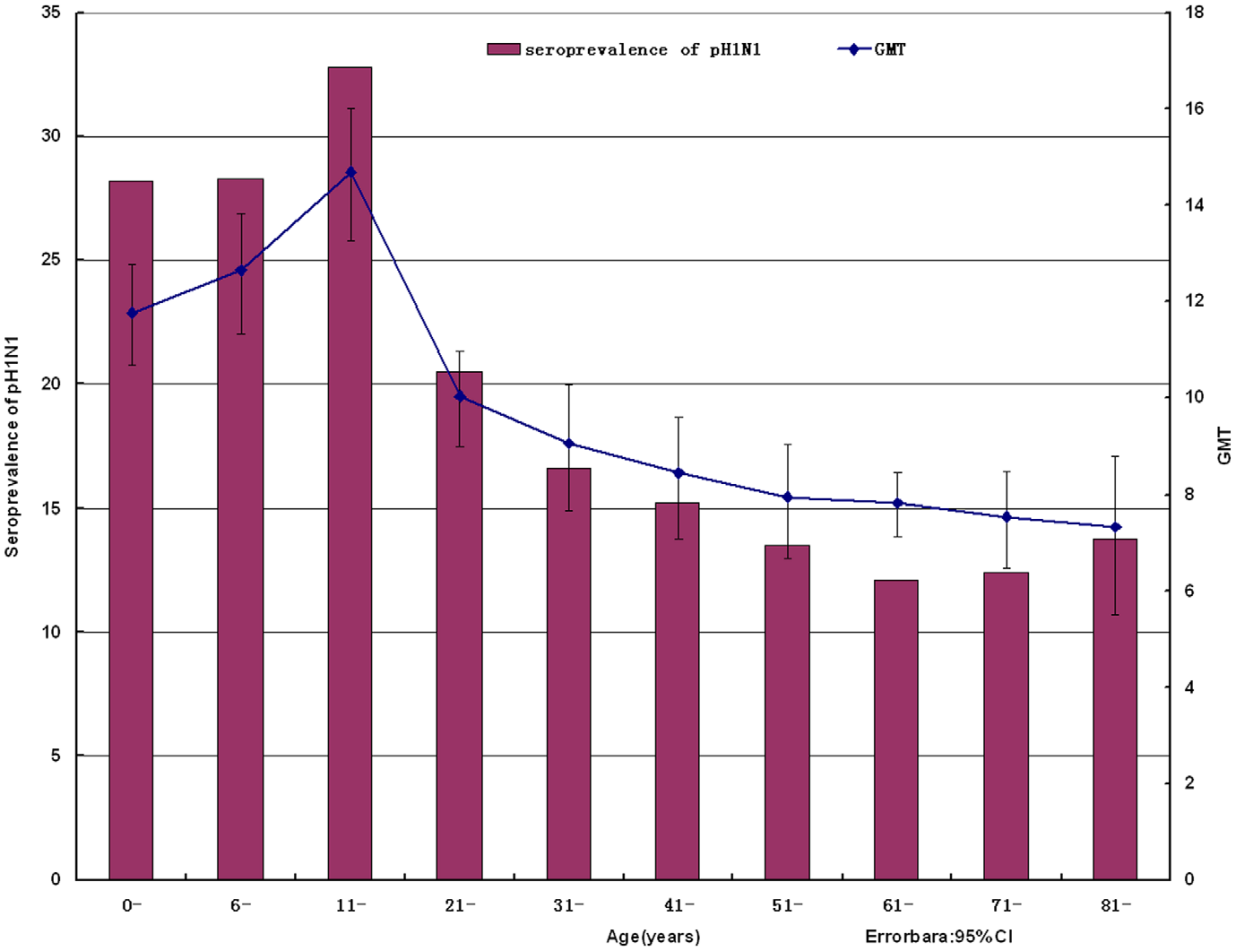
Antibody titers to A/California/7/2009 by HI (hemagglutination inhibition assay). The geometric mean titers (GMTs) and the seroprevalences were plotted according to the age distribution. The 95% confidence intervals (95%CI) for individual HI GMTs were shown as error bars.

Titers to pH1N1 in those under 20 years of age were higher than others. There appeared a sharply rise of GMT, which amounted to 14.7 in 11–20 age group, the highest point among the whole age groups. Then titers fell down steeply and bottomed out in age group of above 80 years, arriving at 7.32. (Figure 1)

**Figure 2:**
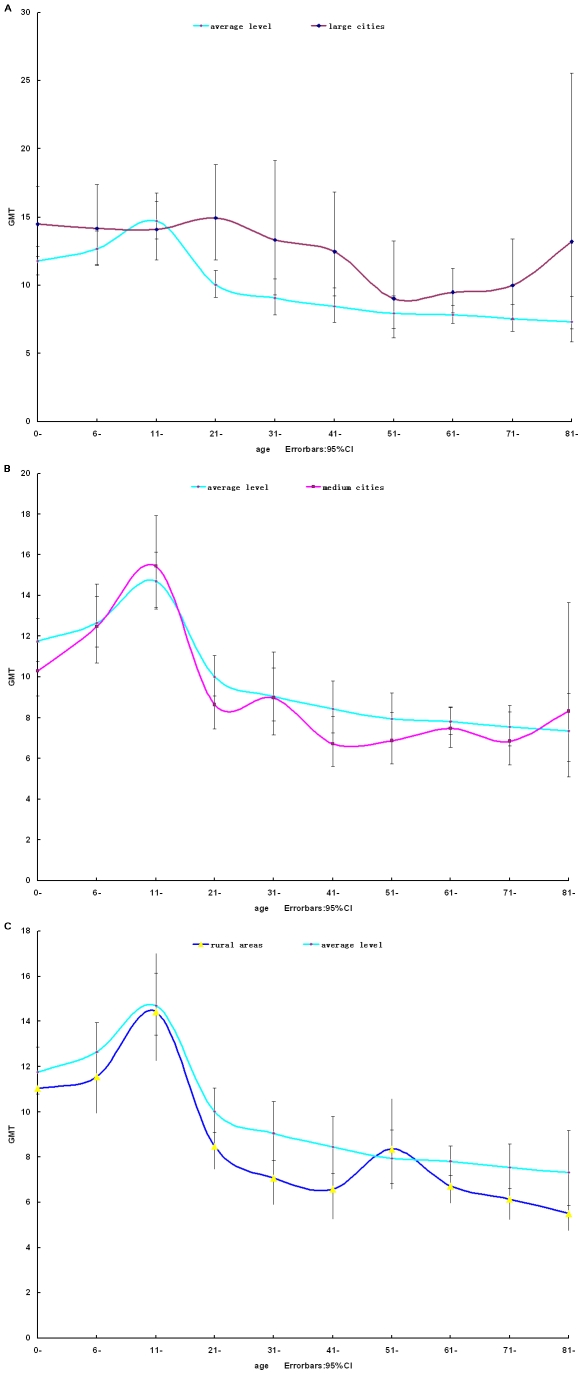
Antibody titers to A/California/7/2009 by HI. Serum samples collected from large cities, medium cities and rural areas, respectively, were tested by HI assay. The GMTs were plotted according to the age distribution. The 95% CI for individual HI GMTs were shown as error bars.

We also compared the antibody titers to pH1N1 in all ten different age groups by region stratum. In large cities (Figure 2A), the GMT of antibodies against pH1N1 are higher than average level of all three region. The GMT leveled off at about 14.3 in under 20 years of age group, followed by a gradually decrease in 21–60 age groups. Beginning at 61–70 age group, especially above 80 years of age, the GMT increased significantly from 9.47 to 13.19.

In medium cities (Figure 2B), the GMT of antibodies against pH1N1 rose rapidly, peaking at just over 15.5 in 11–20 age group, then fell dramatically to 8.6 in 21–30 age group. The GMT fluctuated between just over 6.7 and 8.3 in 31–70 age groups. A slight increase of GMT was also observed in the group of >80 years of age.

In rural areas, as was shown in Figure 2C, similar to the curve of the medium cities, there appeared a peak in 11–20 age group, then the GMT of antibodies against H1N1 dropped gradually, in spite of a slight increase in 51–60 age group. Totally, the GMT of subjects from rural areas was lower than cities.

### 3. Multivariate logistic regression analysis

This regression analysis included 4319 subjects with serum samples and complete data. All variables included were significant in bivariate analysis (Table 1) and remained in the model after stepwise selection. This regression analysis revealed that seroprevalence for pH1N1 was correlated with age and geographic distribution. However, it was irrelative with the symptoms (P>0.05) (Table 3).It was also irrelative with the occupation because which was high correlation with age (P>0.05).

**Table 3:** Multivariate Logistic Regression Model Predicting risk factors for 2009 H1N1 Infection.

Variable	Groups	B	OR(95%CI)	*p*
Region		−0.141	0.869(0.798–0.946)	0.001
Age		−0.016	0.984(0.979–0.990)	0.000
Symptom	Fever	−0.027	0.973(0.669–1.416)	0.887
	ILI	−0.146	0.864(0.587–1.272)	0.458
Occupation		−0.003	0.997(0.934–1.066)	0.940

## Discussion

The influenza A H1N1 2009 pandemic was a challenge to the global public health response. The investigation on epidemiological features was critical for influenza prevention and control. Relying on surveillance of clinical cases can't fully understand the full impact and severity of an epidemic; therefore, the need for more comprehensive serologic survey has emerged. Serologic survey was a useful tool to understand the infection rates and population immunity after natural infection, especially in some populations at high risk, such as elderly adults.

According to our results, the estimate of prevalence after winter wave of the epidemic was 22.82% (985/4,319); adjusted by sociodemographic data which was 18.0% (95%CI, 16.5–19.5%) in Guangdong province. Therefore, the estimate of natural infection population was 17.18 million. We observed the seroprevalence was 20.96% (426/2032) in subjects without any symptoms such as fever, cough, and sore throat. Asymptomatic and mild cases are missed by monitor techniques. The true number of infected cases could not be measured with certainty due to a lack of serological evidence of asymptomatic cases. Our serologic data indicates the importance of asymptomatic cases in transmission of pH1N1, which is helpful for planning surveillance and intervention of future pandemic. On the other hand, it helps explain that seroprevalence for pH1N1 was irrelative with the symptoms in regression analysis (P>0.05).

Our study finds that the infection rate in large cities is much higher than other regions. One possible contributing factor is large cities' strong travel and trade connections to North America and Europe, which facilitate the rapid movement of new influenza virus variants into these areas. More significantly, there is high population density in large cities. Although big cities have better socioeconomic status and medical conditions, the infection rate in rural with low population density is much lower than in cities. This is likely to be explained by strong population mixing and the small geographical extent of these regions. Influenza is a rapidly spreading acute respiratory disease, once the higher likelihood of cases being imported to more densely populated regions, higher speed transmissions happened. These findings resemble those during the 1918 influenza pandemic, when higher infection rates were observed among urban populations than rural populations [9], [10].

Our data suggest that school-age children are most affected by pH1N1 with high infection rates whereas fewer elderly are infected. The seroprevalence of pandemic H1N1 in Guangdong is about 30% in <24 years of age group, and only 12.6% in >60 years of age group. This age-related difference in infection and death rate could not be fully explained by social activity profiles, as some household transmission studies clearly indicated that age is a protective factor in the elderly [11], [12]. Then pre-existing anti –H1N1 immunity is a mechanism to be considered for age-related protection. The hemagglutinin gene of pH1N1 virus is similar to that of viruses that circulated in humans during 1918–1957 [13].To some extent, elderly persons still have cross-reactivity several decades after exposure to 1918 H1N1 virus. Similar epidemiologic observations have been reported in the United States [14] and Canada [15].

Our findings show that in the oldest age groups, especially from large cities, the antibody titers to pH1N1 reached a much higher level, which was likely to be seen in younger age groups. This is likely to be explained that the existence of pre-existing immunity, to some extent, may be affected by the geographical patterns of 1918–1919 pandemic influenza. Gerardo Chowell et al. suggest that higher infection rates were observed among urban populations compared with rural populations in 1918–1919 pandemic [16]. Otherwise antibody acquired through previous exposure in older individuals was further boosted by natural infection of pH1N1 in large cities.

We observed that there was lowest incidence but highest case fatality rate in >60 elderly adults. The concept of original antigenic sin is a probable explanation [17]. Natural infection in humans with antigenically drifted strains of virus induced antibody production against their childhood strains, but response against the current strain was severely diminished. However, inactivated virus induces minimal original antigenic sin, and induces antibody production against the current strain. It suggests that the pH1N1 vaccine inoculation to those elderly people is valuable. On the other hand, immune responses to pathogen infection differ by age [18]. Ageing represents a complex remodeling in which both innate and adaptive immunities deteriorate. Aging affects the humoral and cell mediated immune response qualitatively, as the specificity of the isotypes produced is changed [19], [20]. Therefore, although antibody responses represent the major defense of the organism by neutralizing virus prior to infection, the antibody response of elderly individuals may not be sufficient to adequately prevent a new virus infection. This may have contributed to greater severity if infected in elderly adults.

A series of studies showed that prior receipt of 2008/09 seasonal TIV was partially protective against pandemic influenza infection, while other studies reported an increased risk of pandemic influenza infection following prior receipt of TIV [21], [22]. Our study, however, find no evidence that prior vaccination with TIV significantly altered subsequent risk of pandemic influenza infection in Guangdong, China. This is consistent with the study of Richard et al. in UK [23], who clearly demonstrated that cases were no more or less likely than controls to be vaccinated with season TIV, using a retrospective test-negative case–control study. On the other hand, the pH1N1 virus is antigenically and genetically distinct from recent human seasonal influenza A H1N1 viruses, little or no cross protection would be expected from current seasonal influenza vaccines [24].

In summary, our serological results show a status and pattern of 2009 pandemic H1N1 infections after the winter wave of the pandemic in Guangdong. Our analysis reveals that high-resolution spatial data at the level of age and regions are the key to detecting heterogeneity in influenza transmissibility. Influenza transmissibility results from a complex combination of pre-existing immunity patterns, population mixing and viral strain characteristics, while mortality is also affected by health care and sociodemographic conditions. Our results suggest that the prevalence for pH1N1 is correlated with age and population density. Preexisting antibody may have protected the very elderly from pH1N1 infection, while original antigenic sin and immunosenescence may have contributed to greater severity once infected. Our findings lend support to a vaccination strategy that prioritises high risk groups, especially elderly adults. Our finding can enhance our understanding of the epidemiology of influenza and should be applicable to estimate the possible further wave.

## Methods

### Ethics Statement

Serum samples were collected with written informed consent. The study was approved by the Ethics Committee of Center for Disease Control and Prevention of Guangdong Province(Guangdong CDC) and the Working Group for Influenza Surveillance Network in Guandong, including Guangzhou CDC, Shenzhen CDC, Zhuhai CDC, Shaoguan CDC, Shantou CDC, Foshan CDC, Jiangmen CDC, Zhanjiang CDC, Maoming CDC, Zhaoqing CDC, Huizhou CDC, Meizhou CDC, Shanwei CDC, Heyuan CDC, Yangjiang CDC, Qingyuan CDC, Dongguan CDC, Zhongshan CDC, Chaozhou CDC, Jieyang CDC and Yunfu CDC.

### Study design

We targeted a final sample size of at least 4000, which would have given a power of 90% (with a 2-sided, *p*<0.05) to detect seroprevalence against pH1N1, which was about 20% in China according to sentinel surveillance.

Multi-stage stratified random sampling was introduced with 4319 subjects chosen in Guangdong province. 1500 cases were selected from 5 districts (3 streets were selected in every district) in Guangzhou city, capital of Guangdong, which was representing the large cities. 1500 people were respectively selected from medium city and rural areas, including other 20 cities. (One country or district was selected in every city, at least one street or town was selected in every country or district. Then 1–2 residential area or county was selected in every street or township, respectively.) Every sample was selected in accordance with the principle of random sampling, excluding those who had inoculated with pH1N1 vaccine. All of them were assembled into the following age strata: 0–5, 6–15, 16–24, 25–59, and 60∼. Accompanying information includes collection date, age, gender, job, vaccination, place of residence and symptoms ever occurred in last 6 months.

### Hemagglutination inhibition (HI) assay

HI assay was performed according to the World Health Organization Manual on Animal Influenza Diagnosis and Surveillance [25]. A/California/07/2009 (H1N1) virus (provided by Chinese national influenza center, CNIC) was used as the test virus for HI assay. Sera were pre-treated with receptor-destroying enzyme (RDE, provided by CNIC) at 37°C for 18 h followed by heat-inactivation at 56°C for 30 min, and removal of nonspecific agglutinator by absorbing with the test erythrocytes for 1 hour at 4°C. The treated sera were then serially twofold diluted starting from 1∶10 dilution for HI assay. 0.5% turkey erythrocyte suspension was used for HI assay. Reference/positive control serum (provided by CNIC) with known HI titer, the serum control and back titration of virus antigen were included in each run.

### Statistical analysis

Data were analyzed using SPSS, version 16.0. By convention, seroprevalence was primarily defined as the percentage of serum titers ≥40 by HI [26], [27]. A GMT (geometric mean titers) value of 5 was assigned if HI antibody titer<10. Group GMTs with 95% CIs and proportion with titers≥40 were summarized per study overall and by age stratum. Categorical variables were analyzed using the chi-square test or Fisher exact test. The independent sample *t* test was used to compare GMTs of HI antibodies. Odds ratios that varied significantly by stratum were further evaluated as interaction term in the Multinomial regression model. For all statistical analyses, differences were considered significant when *P*<0.05.
